# Hearing lessons from flies

**DOI:** 10.7554/eLife.19285

**Published:** 2016-08-09

**Authors:** Yi-Nan Lee, Cheng-Ting Chien

**Affiliations:** Institute of Molecular Biology, Academia Sinica, Taipei, Taiwan; Institute of Molecular Biology, Academia Sinica, Taipei, Taiwanctchien@gate.sinica.edu.tw

**Keywords:** Myosin, ubiquitination, hearing, Usher syndrome, MYH9 disorder, genetic screen, *D. melanogaster*, Human, Mouse

## Abstract

Studying the auditory system of the fruit fly can reveal how hearing works in mammals.

**Related research article** Li T, Giagtzoglou N, Eberl D, Nagarkar-Jaiswal S, Cai T, Godt D, Groves AK, Bellen HJ. 2016. The E3 ligase Ubr3 regulates Usher syndrome and MYH9 disorder proteins in the auditory organs of Drosophila and mammals. *eLife* **5**:e15258. doi: 10.7554/eLife.15258

**Image** A montage of scolopidia – the structures that fruit flies require for hearing

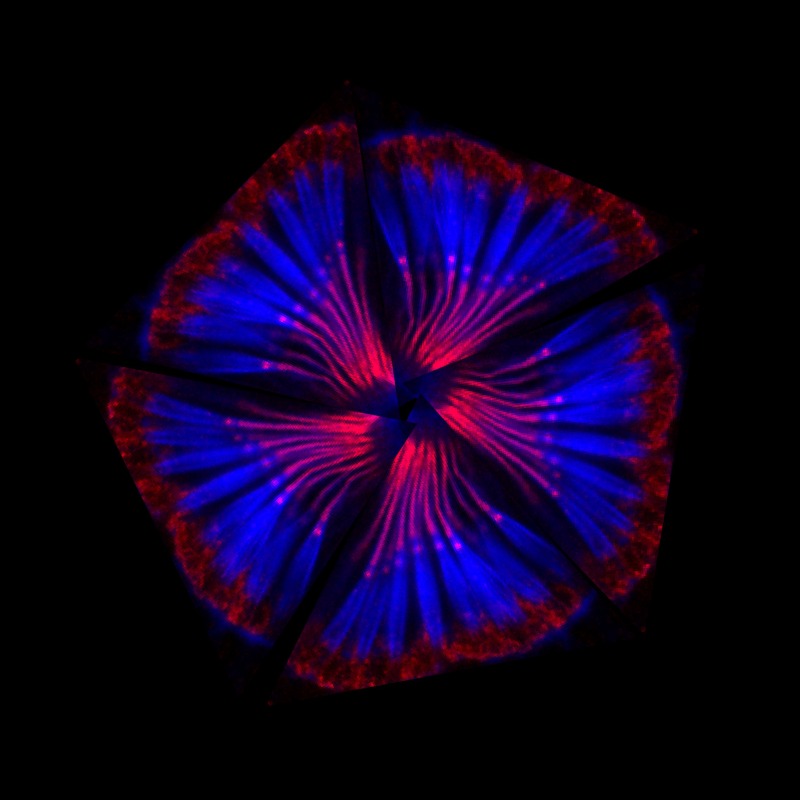

The myosin motor proteins play a variety of roles inside cells, such as transporting cargo around the cell and maintaining the structure of the cell's internal skeleton. Myosins also make important contributions to our sense of hearing, which can be revealed by studying conditions such as Usher syndrome (a severe sensory disorder that causes congenital deafness and late-onset blindness). In humans and other mammals, two myosin proteins called myosin VIIa and myosin IIa have been linked to deafness, but we do not understand how these proteins interact.

Now, in eLife, Andrew Groves, Hugo Bellen and co-workers – including Tongchao Li of Baylor College of Medicine as first author – report evidence of a conserved molecular machinery in the auditory organs of mammals and the fruit fly *Drosophila* (Li et al., 2016). Furthermore, the screen identified an enzyme called Ubr3 that regulates the interaction of the two myosins in *Drosophila*.

Auditory organs convert the mechanical energy in sound waves into electrical signals that can be interpreted by the brain. In mammals, this conversion happens in "hair cells" in the inner ear. These cells have thin protrusions called stereocilia on their surface, and the tips of these stereocilia contain ion channels called MET channels (which is short for mechanoelectrical transduction channels).

Five proteins associated with the most serious form of Usher syndrome – known as USH1 – are key components of the molecular apparatus that enables the MET channels to open and close in response to mechanical force. The USH1 proteins are restricted to the tips of the stereocilia, where they form a complex (Figure 1; Prosser et al., 2008; Weil et al., 1995). Two of the USH1 proteins work together to join the tip of each stereocilium to its next-highest neighbor, forming bundles of stereocilia (Kazmierczak et al., 2007). Deflecting these bundles stretches the stereocilial bundles, which opens the MET channels and triggers the hair cell to produce an electrical signal (Pan and Zhang, 2012). Thus, the USH1 protein complex is essential for maintaining the structural integrity of stereocilia.

**Figure 1.**
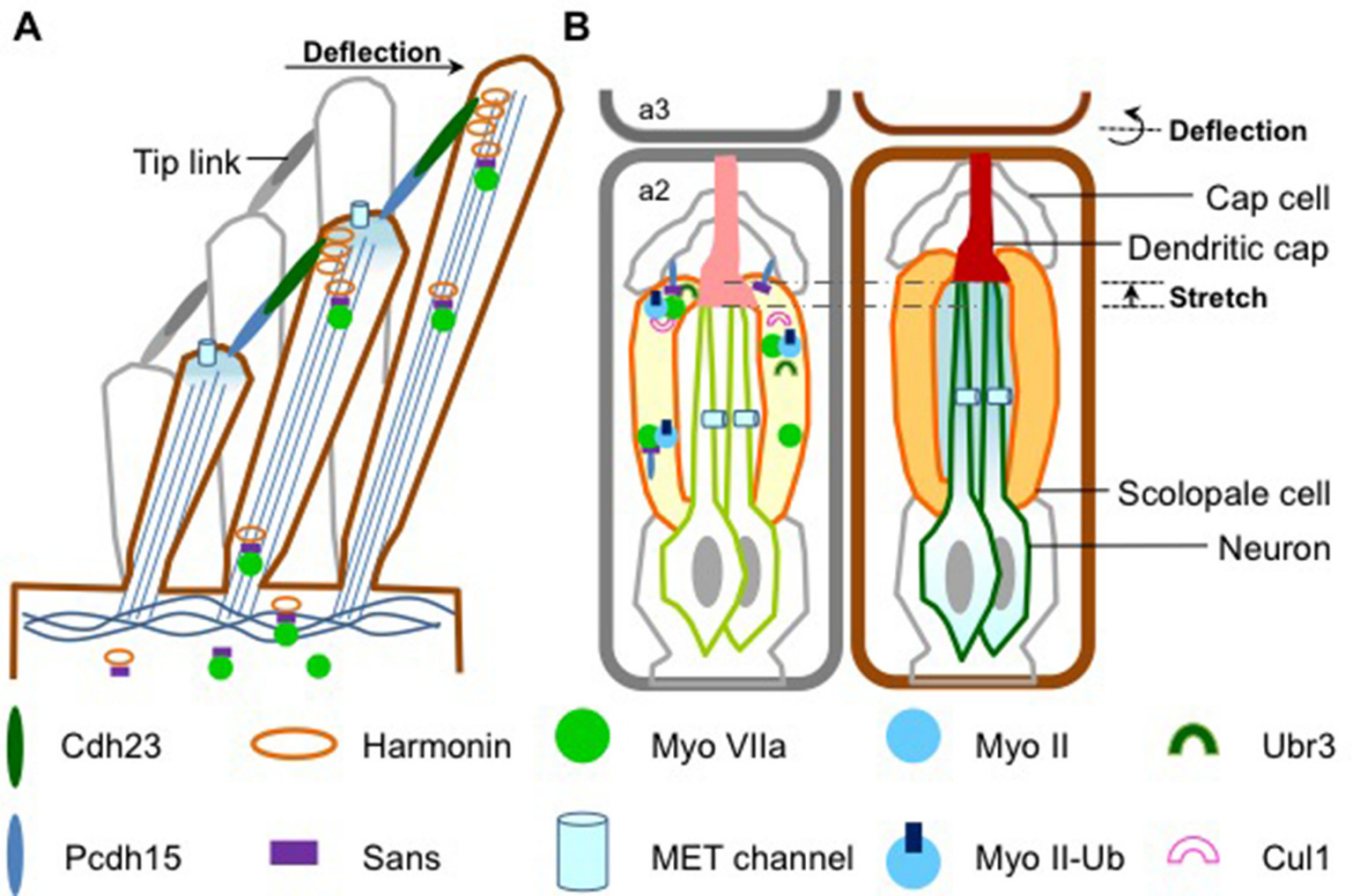
How sound is detected in mammals and *Drosophila*. (**A**) Schematic diagram showing a bundle of three stereocilia protruding from a mammalian hair cell. The deflection of the stereocilia by sound waves results in the opening of the MET channels (pale blue cylinders) and the generation of an electrical signal that travels along sensory neurons to the brain. The motor protein myosin VIIa transports USH1 proteins to maintain the structural integrity of stereocilia. Figure adapted from Figure 1e, Richardson et al. (Richardson et al., 2011). (**B**) Flies use antennae made up of three segments to detect sound. The schematic diagram on the left shows the second segment: there are MET channels for each neuron (outlined in green) and myosin II and myosin VIIa are enriched at the tip of scolopale cells, where USH1 proteins, Ubr3 and Cul1 form a protein complex. A Pcdh15 protein in the USH1 complex anchors the tip of scolopale cell to the cap cell. When a sound wave hits the antenna, the joint between the second and the third segment is deflected (right panel) and the resultant stretching of the second segment opens the MET channels. This depolarizes the sensory neurons, causing them to signal to the brain. Figure adapted from Figure 1b, Boekhoff-Falk and Eberl (Boekhoff-Falk and Eberl, 2014).

Flies do not have ears as such, but they are still able to detect sounds through their antennae. Despite the auditory organs of flies and mammals having different structures, they work in a similar way. In *Drosophila*, structures called scolopidia, which are found suspended in the second segment of the antenna, sense sound vibrations relayed from the third segment (Figure 1). Cells called cap cells and scolopale cells anchor the tip of the scolopidia to the joint between the second and third segments. The scolopale cells also secrete a protein to form the dendritic cap that connects a sensory neuron with the joint. This structure allows the mechanical forces produced by the sound waves to be transmitted to the neuron, activating the MET channels and causing the sensory neuron to produce an electrical signal.

Inactivating the gene that produces myosin VIIa causes the scolopidia to detach from the joint and causes the protein that forms the dendritic cap to be distributed abnormally (Todi et al., 2005; Todi et al., 2008). Now, Li at al. – who are based at Baylor, the Texas Children's Hospital, the University of Iowa and the University of Toronto – show that inactivating the gene that encodes the enzyme Ubr3 has the same effect.

Ubr3 is a type of E3 ubiquitin ligase. These enzymes regulate a number of cell processes by helping to join small proteins called ubiquitins onto other proteins. Using a forward genetic screen, Li et al. found that Ubr3 is enriched in the tips of scolopidia, particularly at the ends of the sensory neurons and in the scolopale cells closest to the joint between the second and third segments.

Li et al. show that Ubr3 and another E3 ubiquitin ligase called Cul1 negatively regulates the addition of a single ubiquitin to myosin II. This means that the loss of Ubr3 increases the rate of the “mono-ubiquitination” of myosin II, which leads to stronger interactions between myosin II and myosin VIIa. Importantly, the mono-ubiquitination of myosin II and the interaction between myosin II and myosin VIIa helps to ensure that they (and also the fly equivalents of Usher proteins) localize correctly to the scolopidial tip. Thus, Ubr3 is crucial for maintaining the structure and function of scolopidia.

Overall, the results presented by Li et al. argue that a conserved model underlies hearing in both *Drosophila* and mammals. In this model, the negative regulation of mono-ubiquitination of myosin IIa (or myosin II in the case of *Drosophila*) by Ubr3 promotes the formation of the myosin IIa-myosin VIIa complex (or the myosin II-myosin VIIa complex in *Drosophila*). The myosin complex then transports the USH1 protein complex to the tips of the stereocilia (or scolopidia) to establish the sound-sensing structure that enables the MET channels to work.

Using the power of fly genetics, Li et al. have identified new components involved in the development and function of auditory organs, and linked them to genes known to play a role in human deafness. Undoubtedly, future studies of these deafness-related genes in the *Drosophila* auditory organ will bring more insights into the interplay among the molecules, including the USH1 proteins, that are important for hearing.
